# POLG1 mutations and stroke like episodes: a distinct clinical entity rather than an atypical MELAS syndrome

**DOI:** 10.1186/1471-2377-13-8

**Published:** 2013-01-15

**Authors:** Antonella Cheldi, Dario Ronchi, Andreina Bordoni, Bianca Bordo, Silvia Lanfranconi, Maria Grazia Bellotti, Stefania Corti, Valeria Lucchini, Monica Sciacco, Maurizio Moggio, Pierluigi Baron, Giacomo Pietro Comi, Antonio Colombo, Anna Bersano

**Affiliations:** 1 Neurological Unit, Ospedale di Desio, Azienda Ospedaliera di Desio e Vimercate, Monza, Italy; 2 Dino Ferrari Center, Neuroscience Section, Department of Pathophysiology and Transplantation (DEPT), University of Milan, Neurology Unit, IRCCS Foundation Ca’ Granda Ospedale Maggiore Policlinico, Milan, 20122, Italy; 3 Neuromuscular Unit, IRCCS Foundation Ca’ Granda Ospedale Maggiore Policlinico, Dino Ferrari Center, University of Milan, Milan, 20122, Italy; 4 Cerebrovascular Unit, IRCCS Foundation Neurological Institute ‘C.Besta’, Via Celoria 23, 20135, Milan, Italy

**Keywords:** POLG1, MELAS, Red-ragged fibers, Stroke-like

## Abstract

**Background:**

*POLG1* mutations have been associated with MELAS-like phenotypes. However given several clinical differences it is unknown whether *POLG1* mutations are possible causes of MELAS or give raise to a distinct clinical and genetic entity, named *POLG1*-associated encephalopathy.

**Case presentation:**

We describe a 74 years old man carrying *POLG1* mutations presenting with strokes, myopathy and ragged red fibers with some atypical aspects for MELAS such as late onset, lack of cerebral calcification and presence of frontal and occipital MRI lesions better consistent with the POLG associated-encephalopathy spectrum.

**Conclusion:**

The lack of available data hampers a definite diagnosis in our patient as well as makes it difficult to compare MELAS, which is a clearly defined clinical syndrome, with *POLG1*-associated encephalopathy, which is so far a purely molecularly defined syndrome with a quite heterogeneous clinical picture. However, the present report contributes to expand the phenotypic spectrum of *POLG1* mutations underlining the importance of searching *POLG1* mutations in patients with mitochondrial signs and MELAS like phenotypes but negative for common mtDNA mutations.

## Background

Mitochondrial myopathy, encephalopathy, lactic acidosis and stroke-like episodes (MELAS) syndrome is a phenotypically and genetically heterogeneous mitochondrial disorder. Stroke-like episodes, which are usually transient and not-disabling, represent the clinical hallmarks. Additional features include seizures, cognitive decline, psychosis, lactic acidosis, migraine, visual impairment, hearing loss, short stature, diabetes and myopathy. MRI shows hyperintensities on T2-weighted and DWI sequences mostly over the temporal, parietal and occipital regions, not confined to a vascular territory. Muscle biopsy typically shows ragged-red and COX-negative fibers, SDH hyperreactivity and, at ultrastructural level, abnormally shaped mitochondria with paracristalline inclusions. MELAS results in 80% of cases from a point mutation, m.3243A>G in the mitochondrial tRNA^Leu(UUR)^ gene (MTTL1) [1,2]. Other mitochondrial DNA (mtDNA) mutations in *MTTL1* gene and other transfer RNA genes (*MTTF*, *MTTV*, *MTTQ*) as well as mutations in other subunits of complex 1 such as MTND1, MTND5 and MTND6 have been also identified as cause of MELAS [3-6]. Indeed, mutations in nuclear genes leading to secondary mtDNA changes (depletions and multiple deletions), have been described as emerging causes of MELAS [7]. Recently, Deschauer et al. described a patient showing stroke-like episodes and a right occipital lesion, headache, seizures, elevated CSF lactate, ragged–red fibers and carrying heterozygous mutations in mtDNA polymerase gene (*POLG1*) arguing that MELAS could be included in *POLG1* spectrum phenotype [7]. *POLG1* mutations were described, so far, in families with autosomal dominant and recessive chronic progressive external ophthalmoplegia (PEO), Alpers syndrome, parkinsonism, optic neuritis and late onset ataxia [8,9]. We previously reported a cohort of 67 patients affected by myopathy with or without PEO, in which 19.4% of patients carried *POLG1* mutations [10]. Herein, we report the 2-year neurological follow up of one of these patients disclosing over time a clinical phenotype highly consistent with MELAS.

## Case presentation

### Patient’s history

**Figure 1:**
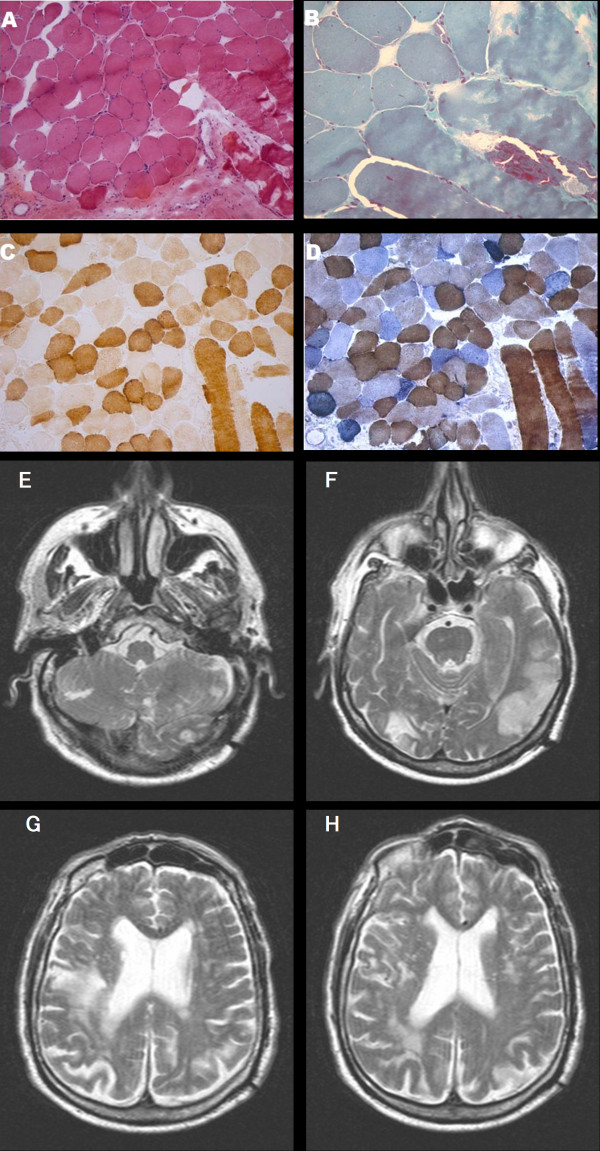
**A-D: Skeletal muscle biopsy showing one ragged red fiber with histological methods (A-B: H&E, 10X ; B: GT, 40X).** Histochemical reactions for COX (**C**, 10X) and COX-SDH (**D**, 10X) in cross serial sections show lack of COX activity in several skeletal muscle fibers (**C**), many of which also show increased SDH activity (**D**).**E-H**: Axial T2-weighted cerebral MRI sequences showing a cortico-subcortical fronto-parietal hyperintensity with restricted diffusion (not shown) consistent with acute ischaemic lesion and bilateral old ischaemic lesions in the right occipital and left temporal lobe.

A 74 year old man was admitted to the Neurological Unit of Desio Hospital in September 2004 for sudden onset of a speech disorder and left side weakness. Over the past ten years he developed a progressive bilateral ptosis, hearing loss and difficulties in swallowing. For these symptoms he was hospitalized in 1996 elsewhere. The neurological examination performed at that time revealed bilateral ptosis, bilateral hearing loss and mild dysphagia. The neurophysiological examination, performed in 1996, revealed a myopathic pattern. However, at that time the patient refused any further investigation including muscle biopsy. The remaining patient’s past history was unremarkable. In particular, except for a mild increase in cholesterol levels, cerebrovascular risk factors were absent. Family history was negative for muscular disorders, stroke, hearing loss, diabetes, short stature, headache, mental retardation or dementia. Neurological examination, performed on admission in 2004, revealed bilateral ptosis and severe ophthalmoparesis, hearing loss, dysarthria, left facial nerve palsy and mild left side hemiparesis. Acute phase NIHSS score was 4. Fatigability and myotonic phenomena were absent. Acute phase cerebral CT scan was negative for acute ischemic lesions. Cerebral MRI, performed two days later, showed a right pre-rolandic hyperintensity on T2-weighted sequence, not confined to a specific vascular territory, with a slight enhancement after gadolinium, consistent with an acute ischaemic lesion. Other bilateral focal hyperintensities, mostly in the posterior circulation territory (cerebellar bilateral, left temporo-occipital and frontal) consistent with stabilized ischaemic lesions, were also found (Figure 1). Biochemistry was normal except for increased level of serum creatine kinase (189→643, n.v 38–174 U/l). Inflammatory and autoimmune markers were normal as well as lactate serum level (1,7; n.v 0.5-1.8 mmol/l). Electrocardiogram, echocardiography and abdominal ultrasonography were negative. Audiometry revealed a moderate sensorimotor bilateral hypoacusia. Ophthalmological examination, including fluoroangiography, excluded a pigmentary retinopathy. Electroencephalography was normal too. Neurophysiological examination demonstrated myopathic signs in frontal, masseters, orbicularis oculi and mouth muscles without denervation and normal findings in the other examined muscles (right biceps, triceps, interosseous and left and right quadriceps, tibialis anterior, medial gemellus). No signs of polyneuropathy were detected. Ticlopidine 250 mg twice daily was started. Left hemiparesis resolved completely within 7 days. During the following years a progressive worsening of ptosis, ophthalmoparesis, hearing loss and dysphagia was observed. Cognitive impairment was not detected at MMSE. Headache and seizures were not referred too. In November 2008 the patient was hospitalized again for a sudden onset of aphasia. No other new-onset neurological symptoms were referred. The neurological examination performed at that time showed global aphasia and a right hemiparesis with right Babinski sign. The NIHSS score was 4. Residual signs such as severe bilateral ptosis, bilateral opthalmoparesis, bilateral hearing loss, dysarthria, solid food dysphagia were unchanged. Fatigability, cerebellar ataxia or sensitive deficits were absent. Biochemistry, echocardiography, epiaortic vessel Doppler ultrasound were normal. Cerebral CT scan performed in the acute phase was unmodified in comparison to the previous ones. Cerebral MRI at day 3 demonstrated a small cortical-subcortical left temporo-occipital lesion with DWI restriction (Figure 1). The strength deficit resolved in a few days whereas a language rehabilitation was necessary. Aphasia progressively recovered and was not present at the three months follow-up. The patient continued to assume ticlopidine.

### Genetic analysis

**Figure 2:**
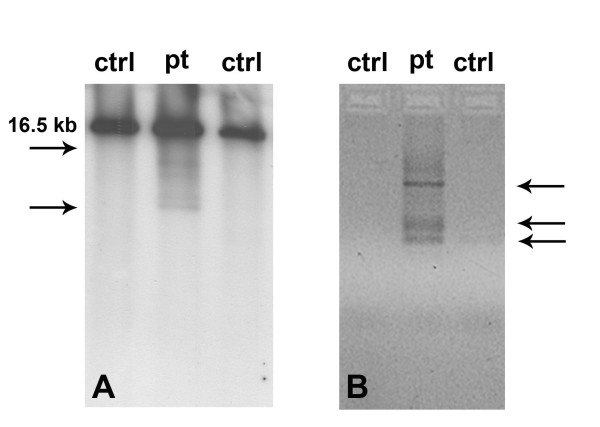
**Molecular analysis of muscle-derived mitochondrial DNA.** Southern blot (**A**) and PCR assay (**B**) showing the accumulation of multiple deletions in patient’s tissue.

Total DNA was extracted from peripheral blood and muscle. PCR-RFLP analysis did not reveal m.3243A>G mutation. Southern blot analysis of muscle mtDNA [11] revealed the presence of several bands compatible with mtDNA multiple deletions (Figure 2). A specific PCR assay identified mtDNA multiple deletions using two primers (forward 7440–7465 and reverse complement 16135–16110) and the following amplification protocol: an initial denaturation at 94°C for 2 min, followed by 25 cycles (94°C for 30 s, 55°C for 30 s, and 68°C for 90 s) and a final extension for 2 min at 72°C (Platinum HiFi Taq Polymerase by Invitrogen, Carlsbad, CA). The entire coding sequence of POLG1 gene (NM_002693.1) was PCR-amplified and directly sequenced disclosing the presence of three heterozygous variants: the c.752C>T (exon 3, p.T251I), c.1760C>T (exon 10, p.P587L) and c.3556G>C (exon 22, p.D1186H). We did not test any other family member for POLG mutations since parents are not alive and siblings are not available. Both the mutations p.T251I and p.P587L were reported several times in subjects showing multiple clinical phenotypes, according to the Human DNA Polymerase Gamma Mutation Database (http://tools.niehs.nih.gov/polg/). The missense mutation c.3556G>C was not listed in the dbSNP database (http://www.ncbi.nlm.nih.gov/projects/SNP/) as well as in the POLG mutation database and it was not found in more than 200 Italian healthy controls. The affected residue (position 1186) is evolutionarily conserved across species. Three software programs were used to predict the overall severity of this variant. According PolyPhen-2 (http://genetics.bwh.harvard.edu/pph2/), SIFT (Sorting Intolerant From Tolerant, http://sift.bii.a-star.edu.sg/) and PMut (http://mmb.pcb.ub.es/PMut/), the p.D1186H change is predicted to be highly deleterious.

### Skeletal muscle and nerve histopathology

In 2004 the patient underwent a left biceps skeletal muscle biopsy. Cryostatic cross sections were processed according to standard histological and histochemical techniques including Haematoxylin & Eosin (H&E), Gomori Trichrome (GT), Cytochrome c Oxidase (COX), Succinate Dehydrogenase (SDH) and double reaction for COX and SDH [12]. Electron microscopy studies were performed as described [13].

Histological examination of muscle specimen showed slight fiber size variability, some nuclear centralizations and several fiber splittings along with a consistent number (n=10) of ragged red fibers (RRF). Necrosis was observed in a few fibers. Histochemically, several fibers were Cytochrome c Oxidase (COX)-negative, many of these fibers were also intensely SDH-positive (Figure 1).

DNA sample and muscle biopsy were collected after obtaining patient informed consent according to the local ethics and privacy and human subjects’ protection regulations and were taken as part of standard patient care.

## Conclusion

The human mitochondrial genome is replicated by the DNA polymerase γ, pol γ, which is encoded by *POLG1*, which is a 23 exons nuclear gene located on chromosome 15q25. Heterozygous and homozygous *POLG1* mutations have been typically associated with heterogeneous and severe clinical phenotypes of PEO, both in autosomal dominant or recessive form. They can also result in adult onset cerebellar ataxia with mtDNA multiple deletions and Alpers syndrome, an autosomal recessive hepatocerebral disease characterized by severe developmental delay, intractable seizures, liver failure and death in childhood. Moreover, throughout the years a wide spectrum of clinical findings including parkinsonism, neuropathy, optic neuritis, psychiatric disorders has been described in *POLG1* mutations carriers [12,14-19]. Deschauer et al. 2007 described a patient presenting with occipital seizures and residual homonymous hemianopsia, headache and ataxia and carrying two heterozygous *POLG1* mutations [7]. The presence of occipital symptoms and lesions, interpreted as stroke-like episodes, together with elevated CSF lactate and ragged red fibers, posed the suspicion of MELAS, raising the question whether *POLG1* mutations could be associated with a MELAS like phenotype [7,20]. However, given several clinical differences between *POLG1* patient characteristics and MELAS, it has been supposed that, despite some overlapping symptoms, *POLG1* represent a distinct clinical and genetic entity. POLG-associated encephalopathy has been postulated to give rise to a distinct phenotype, including variable age at onset, either recessive or dominant inheritance pattern and peculiar neuroimaging findings characterized by predominant posterior ischemic lesions and lack of cerebral calcification [21].

Our case had some atypical aspects for MELAS such as late onset, lack of cerebral calcification and presence of frontal and occipital MRI lesions better consistent with the POLG associated-encephalopathy spectrum. However, it is difficult to assess with certainty whether our patient could be classified as MELAS-like or POLG-associated encephalopathy. Moreover, although the patient did not present any vascular risk factor, given the elderly age a co-incidence between a POLG myopathy and a cerebrovascular disease can not be excluded at all.

However, it is difficult to compare MELAS, which is a clearly defined clinical syndrome, with *POLG1*-associated encephalopathy, which is so far a purely molecularly defined syndrome with a quite heterogeneous clinical picture. In addition, although 140 POLG mutations have been described in patients with symptoms that suggest mitochondrial disease, most of mutations are reported in heterozygous in whom each POLG allele can be one or more different mutations and only few of these has been replicated in not related families. This makes difficult a clear definition of phenotype and in providing evidence of the disease causing nature of these mutations [22].

Our patient harboured the haplotype p.[T251I P587L] in combination with D1186H located in polymerase domain. The haplotype T251I and P587L has been already described but it is currently not possible to know whether T251I or P587L is the primary pathogenic allele or whether both mutations are necessary to cause disease. Nevertheless both variants have also been reported in trans one each other in affected subjects. Notably both P587 and D1186 residues are located in the DNA binding channel of POLG enzyme. Thus, the substitutions found in our patient could be consistent with a reduced DNA binding capacity.

However, given the lack of available data, the implementation of existing databases (http://tools.niehs.nih.gov/polg/) including single unpublished observations from clinicians, is necessary to determine allelic frequencies of the myriad of *POLG1* mutations [22,23]. Ongoing studies on biochemical measurements of polymerase activity and in vivo measurement of mitochondrial dysfunction will provide further issue on their pathogenic role.

The present report contributes to expand the phenotypic spectrum of *POLG1* mutations underlining the importance of searching *POLG1* mutations in patients with mitochondrial signs and MELAS like phenotypes but negative for common mtDNA mutations.

## Informed consent

Written informed consent was obtained from the patient for the publication of this case report and any accompanying images.

## Competing interest

All the authors disclose any conflicts of interest including any financial (grant or fundings), personal or other relationships with other people or organizations within three years of beginning the work submitted that could inappropriately influence or bias their work.

## Authors’ contributions

AC, BB, MGB have made substantial contributions to case conception and design, acquisition of data and have been directly involved in drafting and revising the manuscript. AB, MS, VL, MM, AC have made substantial contributions to genetic, biochemical and hystological analyses and interpretation of data. DR, SL, SL PL, GPC, AB (*) have made substantial contributions case to conception and design, analysis and interpretation of data and have been directly involved in drafting and revising the manuscript critically for important intellectual content. All authors read and approved the final manuscript.

## Pre-publication history

The pre-publication history for this paper can be accessed here:

http://www.biomedcentral.com/1471-2377/13/8/prepub
